# Urban Health Extension Services Utilization in Bishoftu Town, Oromia Regional State, Central Ethiopia

**DOI:** 10.1186/s12913-017-2129-z

**Published:** 2017-03-14

**Authors:** Ewunetu Aberra Gebreegziabher, Feleke Hailemichael Astawesegn, Antehun Alemayehu Anjulo, Mirkuzie Woldie Kerie

**Affiliations:** 1 Kellem Wollega Zonal Health Department, Dembidolo, Ethiopia; 2 School of Public Health, College of Health sciences and Medicine, Wolaita Sodo University, Wolaita Sodo, Ethiopia; 3 School of Medicine, College of Health sciences and Medicine, Wolaita Sodo University, P.O. Box: 138, Wolaita Sodo, Ethiopia; 4 Department of Health policy and Management, Jimma University, Jimma, Ethiopia

**Keywords:** Urban health extension, Urban health extension service utilization, Model household

## Abstract

**Background:**

Ethiopia has been deploying specially trained new cadres of community based health workers in urban areas of the country known as urban health extension professionals since 2009. At present, relatively little work has focused on understanding to what extent this new program is accepted and used by the community.

**Methods:**

Both qualitative and quantitative surveys were performed from March 10, 2012 to March 25, 2012 to explore the utilization of urban health extension services in Bishoftu Town, Oromia regional state, Central Ethiopia using a cross sectional study design. Qualitative data were collected using a total of 4 focus group discussions and 26 in-depth interviews. Quantitative data were collected from 418 randomly selected households using pre-tested, structured, interviewer-administered questionnaires. Data entry and analysis were done using SPSS version 16.0. Qualitative data were analyzed thematically.

**Results:**

Of the 418 interviewed households, 72.8% of them had at least one service related contact with urban health extension professionals in the previous 6 month. The mean frequency of service related contact with Urban Health Extension Professionals was found to be 2.24 (±1) contacts per 6 months. The total number of households graduated as a model family in the study area was 3974 (14.3%). Though participants felt that urban health extension professionals faced community resistance at program implementation, its acceptability greatly improved in this study. Despite this, individual competencies of urban health extension professionals, availability of supply and logistic system, and the level of support from kebele officials were reported to influence the program acceptability and utilization.

**Conclusions:**

The introduction of urban health extension professionals positively changed the attitude of the majority of the households involved and improved the acceptability of the program. All stake holders, governmental and nongovernmental organizations, should have supportive systems to increase the acceptability and utilization of urban health extension services.

**Electronic supplementary material:**

The online version of this article (doi:10.1186/s12913-017-2129-z) contains supplementary material, which is available to authorized users.

## Background

Ethiopia is the country with the largest population in Africa. The increase in urban population density of Ethiopian cities is a direct result of the expansion of the cities, followed by the movement of people from rural areas to cities. This urged the Ethiopian government to introduce innovative community based programs in order to promote health, prevent diseases and increase access to the treatment of communicable diseases in the urban area. The urban health extension program is organized as a component of other urban health services and aims to create a healthy community, a healthy living environment and a healthy work place. They plan to accomplish this, using female nurse professional trained on health extension program to achieve the principles of Primary Health Care (PHC) [1]. PHC is the key to achieve an acceptable level of health throughout the world with full community involvement. Therefore many national health systems, based on PHC, undertook a major reform in health services in order to achieve their aims [2, 3]. Likewise, the Ethiopian government has introduced an innovative health service delivery system through the implementation of the Health Service Extension Programme (HEP) [4, 5].

HEP is a package of basic and essential promotive, preventive and curative services for selected diseases. It is designed based on the principles of PHC to improve the health status of families and households, with their full participation and using local technologies. HEP increases the coverage of PHC services, mainly by producing model households using model family training. This program involves front-line community health workers. They are called Health Extension Workers (HEWs) and they are providing care for the community focussing on four areas: disease prevention and control, family health, hygiene and environmental sanitation and health education across the country. The model family training comprises a total of 96 h of training on basic hygiene and environmental sanitation (30 h), family health care (42 h), and disease prevention and control (24 h). Households which attend at least 75% of the training and implement at least 75% of the HEP packages receive certificates of completion at a graduation ceremony and graduate as model households (families) [6–8].

Ethiopian Ministry of Health’s has been aggressively producing and assigneda variety of community health agents called volunteer Community Health Workers (CHWs). CHWs consist of frontline public health workers who are selected, trained and working for the communities living in the place where they came from. These include Trained Traditional Birth Attendants, Community Based Reproductive Health Agents and Community Health Agents.

The HEP is designed to improve access and fair distribution of health care services focusing on sustained preventive health actions and increased health awareness. Every health post will have at least two HEWs, who have undergone a 1 year training course [9, 10].

As per the guideline of HEW, they are expected to spend 75% of their time in undertaking outreach activities at the kebele level. They go from house to house in their respective kebele (neighborhood). The rest of their time they stationed at the local health post. All HEWs have completed high school and received additional training for 1 year at an undergraduate level. The HEWs are different from the volunteer CHWs. They are government employees and get a monthly salary, while CHWs are volunteers. HEWs receive more advanced, comprehensive training in comparison to volunteer CHWs [9].

HEP was initiated in Ethiopia in order to promote prevention and control activities (in four areas of care: disease prevention and control, family health, hygiene and environmental sanitation and health education). They are primarily focused on the rural population of Ethiopia where more than 85% of the population resides and where there are many community members suffering with communicable diseases [12]. However, several studies revealed that in a number of African countries there is no significant difference between under-five mortality in urban and rural areas. In addition, urban areas have a poor health status indicator [9, 11, 12].

In most African countries, the urban poor are exposed to environmental risks and preventable, life threatening diseases. Existing environmental infrastructure is often inadequate to provide clean drinking water or to hygienically treat household liquid and solid wastes. This situation endangers the health and productivity of the urban poor, especially women and children [9, 10, 13].

Like many developing nations, due to the high prevalence of communicable diseases in the general Ethiopian population, there are high of morbidity and mortality rates. Poor access to health services and complex health systems in Ethiopia have contributed to the high burden of disease and high rate of mortality in the country [14–16].

Ethiopian cities are also the fastest growing communities in the country, adding 4.2% to the overall population per annum [17]. It is a well known fact that such rapid urban growth is often accompanied by the spontaneous appearance of settlements at the cities’ peripheries and by a disorganization of these settlements with insufficiently developed infrastructure, e.g. poorly planned housing, utilities and streets [18, 19].

Upon recognition of this growth in the face of the challenges mentioned and to improve the public health interventions, the Federal Ministry of Health (FMOH) started implementing HEP in urban areas since 2009 [20–22].

Taking advantage of the lessons learned and the successes of the rural HEP, the FMOH launched the Urban Health Extension Program (UHEP) in urban kebeles (neighbourhoods) of the country since 2009 [1].

Urban Health Extension Professionals are trained nurses placed in urban health posts to bridge households, communities and health facilities at the periphery. Each health center serves 40,000 people; therefore, one urban health extension professionals is assigned to 500 households [21].

Since the start of the implementation of the new UHEP, there has not been any study done to examine how the Urban Health Extension Services (UHES) program was accepted and used by the community. This study intends to explore utilization of urban health extension services in Bishoftu Town, Oromia Regional State, Central Ethiopia. It may serve asbaseline for program implementers, decision makers and future researches in this area.

## Methods

### Study area and period

A community based, descriptive, cross sectional study was carried out from March 10, 2012 to March 25, 2012 in Bishoftu town of the Oromia Regional state. This study employed both qualitative and quantitative methods.

The town is located 45 km south east of Addis Ababa, the capital city of Ethiopia. According to 2007 census report of Ethiopia, the total population of the town was 100,114 people of which men constitute 47,938 (47.8%) and women constitute 52,176 (52.2%). It lies 9^0^ N latitude and 40^0^E longitude at an altitude of 1950 m above sea level. The average maximum and minimum temperature of the area is 34.7 °C and 8.5 °C respectively, and average relative humidity is 61.3%. The rainfall is bimodal. It receives an annual rainfall of 1151.6 mm of which 84% is received during the long rainy season covering June to September and the remaining in the short rainy season extending from March to May.

In the town there are two hospitals and three government health centers providing health services to the community. The town is divided into nine administrative kebeles and 6 ketenas. All the nine kebeles in the town started implementing the urban health extension program since 8/12/2009.

### Sample size

The sample size for quantitative data were determined to achieve a 95% confidence interval. We assumed 50% of the proportion of graduated model households would be involved with a 5% of margin of error and a 10% o non-response rate. The calculated sample size for the household survey was 423.

In addition to four focus group discussions, the compilation of participants included 36 Urban Health Extension Professionals discussants divided in four groups, 2 representatives of kebele health committees and additional community members. Twenty-six in-depth interviews were performed with 9 key informants to collect our qualitative data. The interviewees included Urban Health Extension Professionals, 3 supervisors, 3 health centre heads, 9 kebele leaders and 1 head of the town health office.

### Data collection and sampling procedure

For the quantitative aspect, a face-to-face interview of household members was conducted using a structured interviewer administered questionnaire. The inquiry had questions on basic socio-demographic characteristics, household socioeconomic status and other important study variables (Additional file 1).

All nine kebeles in Bishoftu town were included in the household survey. To create a representative sample, each kebele was given the appropriate number of interviews based on the numerical proportion of household in that particular kebele. A systematic random sampling technique was employed to select study participants. A list of all the households in each kebele was obtained from the kebele administration office and one respondent was included from every selected household. Index case was selected and interviewed using lottery method when more than one eligible respondent present in a house. The principle investigators and ten investigators were involved in data collection.

Data on qualitative information were collected using Focus Group Discussion (FGD) and in-depth interview guides (Additional file 1). Four focus sessions of nine participants each were carried out with Urban Health Extension Professionals, kebele health committee members and community members over the study period. The investigators moderated the group discussion and took detailed notes. The FGD in each group took about one and half to 2 h. In addition to focus group sessions, 26 in-depth interviews were conducted with Urban Health Extension Professionals, health extension program supervisors, kebele administrators, health centre heads and the head of town health office. The main investigator conducted the interviews and wrote detailed notes. Each in-depth interview lasted approximately one to one and half hours. Participants were identified and interviewed purposefully. To be included in the study, the participant had to live in the area for at least 6 months and that participant had to be either the female head of the household or spouse of the head of the household.

### Data processing and analysis

Each questionnaire was checked for completeness, and then data were entered into the database, then cleaned and explored for missing values or any other inconsistencies. Analysis was conducted using SPSS version 16. Descriptive statistics including frequency tables, graphs and descriptive summaries were used to describe the study variables. For the qualitative data, the data was transcribed and then translated into English. Similar responses were grouped and summarized based on thematic area or key variables. Finally, results of the qualitative study were presented in narratives triangulated with the quantitative results.

## Results

### Socio-demographic characteristics of respondents

Four hundred eighteen households were interviewed giving a response rate of 98.81%. The mean age of the respondents was 39.6 years. One hundred thirty eight (3.0%) of respondents attended primary school education, while 99 (23.7%) could read and write without formal education. Regarding the ethnic origin, the majority 188 (45%) was Oromo by ethnicity, followed by Amhara 144 (27.3%). Marital status, 344 (82.3%) of the interviewees were married, 43 (10.3%) were single. By religion, more than half, 242 (57.9%) were Orthodox Christian followers and 93 (22.2%) were followers of Islamic religion. By occupational status 171 (40.9%) were housewife’s and 136 (32.5%) were employed (Table 1).

**Table 1:** Percentage distribution of respondents by selected individual characteristics in Bishoftu town, central Ethiopia, March 2012 (*n* = 418)

Socio-demographic characteristics	No. of respondents	Percentage (%)
Status of respondent in the family		
Household head	74	17.7
Spouse	344	82.3
Sex		
Male	96	22.97
Female	322	77.03
Age (year)		
18–24	29	6.9
25–35	147	35.2
36–45	124	29.7
46–55	76	18.2
55+	42	10.0
Mean age in years (± SD) ^a^	39.6 (±12)	
Marital Status		
Single	43	10.3
Married	344	82.3
Widowed	10	2.4
Divorced	21	5.0
Educational level		
Illiterate (cannot read or write)	58	13.9
Write and read (no formal education)	99	23.7
1–8	138	33.0
9–12	95	22.7
Diploma & above	28	6.7
Ethnicity		
Oromo	188	45.0
Amhara	114	27.3
Gurage	63	15.1
Tigray	29	6.9
Others ^b^	24	5.7
Religion		
Orthodox	242	57.9
Muslim	93	22.2
Protestant	70	16.7
Others ^c^	13	3.2
Occupation		
Housewife	171	40.9
Employee	136	32.5
Trader	108	25.8
Others ^d^	3	0.7
Household Size		
1–5	292	69.9
5–9	119	28.5
9+	7	1.6
Mean household size (± SD) ^a^	4.7 (±1.8)	
Monthly income		
150–650	62	14.8
651–1400	170	40.7
1401–2350	134	32.1
2351–3550	48	11.5
3551+	4	1.0
Median household income (± SD) ^a^	1300 (±735.7)	
Monthly expenditure		
150–650	88	21.1
651–1400	200	47.8
1401–2350	107	25.6
2351+	23	5.5
Median household expenditure (± SD) ^a^	1000 (±590.1)	
Self-perceived health status		
very good	82	19.6
good	132	31.6
normal	180	43.1
bad	23	5.5
very bad	2	0.2

^a^ Means with ± standard deviation

^b^ Silte, Welaita, kenbataetc

^c^ Catholic, waqefetaetc

^d^ Student, No occupation

### Utilization of urban health extension services

Three hundred and eight (72.8%) households reported they had service related contact with Urban Health Extension Professionals at least once in the previous 6 months prior to the study period. The mean frequency of service related contact with Urban Health Extension Professionals was found to be 2.24 (±1) contacts per 6 months. Among those who reported a contact, the majority, 279 (90.6%), reported that they were visited by the Urban Health Extension Professionals at their home (Table 2).

**Table 2:** Location of service related contact with urban health extension professionals in Bishoftu town, central Ethiopia, March 2012 (*n* = 418)

Variables	Number of house hold (n)	Percent (%)
Place of contact		
Home	279	66.7
Public places (School/working place)	14	3.3
Health post	11	2.6
Community meetings	4	0.9
Never had contact	110	26.3
Frequency of total contact in the last 6 months		
Five times or more	23	5.5
Four times	85	20.3
Three times	115	27.5
Twice	51	12.2
Once	34	8.1
Never had contact	110	26.3

Data on the use of other health services has also been collected in household surveys to compare how often UHES is used relative to the other existing health services. Two hundred ninety one (69.61%) households had visited other health facilities at least once in the previous 6 months prior to study period.

By categorizing the contents of service related contacts in core UHES areas (disease prevention and control, family health, personal hygiene and environmental sanitation, and first aid and emergency services) the following results were obtained. Model family graduation requests will be presented separately.

Of all the households in the study area, Urban Health Extension Professionals reported that 3974 (14.3%) households had been trained and certified as model households. In comparison, the household survey data indicates that only 99 (23.7%) of the respondent households reported that they had been invited by Urban Health Extension Professionals to participate in model family training. From the 99 households who were asked to participate in model family training, 48 (48.5%) of them were willing to participate in the training and forty (83.3%) of these finished and graduated from their training. On the other hand, four households discontinued the training and four are actively still in training. Fifty one (51.5%) households were not willing to train as a model family. The reasons given by the household respondents included shortage of time 40 (78.4) and lack of interest to train 6 (11.8%).

Urban Health Extension Professionals participants complained most that the urban people work through the week and have no time for participation. Urban Health Extension Professionals tried to solve this problem by giving trainings on weekends, after work hours. All service related contacts included some element of health education (Table 3).

**Table 3:** Themes addressed by the urban health extension professionals in Bishoftu town, Central Ethiopia, March 2012 (*n* = 308)

Themes	Number of house hold (n)	Percent (%)
Personal hygiene and environmental sanitation		
Housing and environmental sanitation	238	77.3
Solid and liquid waste management	194	63.0
Excreta disposal/latrine utilization	181	58.8
Water and food safety	146	47.4
Disease prevention and control		
Prevention of Malaria	107	34.7
Prevention of diarrhoea	73	19.5
Prevention of HIV/AIDS/TB	30	9.7
Non communicable disease	46	15.0
Mental health	59	19.2
Family health		
Vaccination advice	207	67.2
Nutrition counselling	60	19.4
Family planning	59	19.2
Pregnancy and delivery care	42	13.6
Adolescent reproductive health	24	7.8

Discussions were conducted with Urban Health Extension Professionals to explore the reasons why some topics are covered in some households, but not in others. The discussions indicate that the Urban Health Extension Professionals select the topics based on their perceived assessment of the household’s need.

In all of the kebeles, Urban Health Extension Professionals reported they were delivering health education related to disease prevention (both communicable and non-communicable). However, for HIV/AIDS they provided services in addition to disease prevention including HIV/AIDS counselling and testing.

From 308 households who had contacted Urban Health Extension Professionals in the previous 6 months, 205 (66.55%) reported receiving health education and/or advice related to disease prevention and control. Urban Health Extension Professionals indicate that the knowledge and behavioural practices of households towards prevention of both communicable and non-communicable disease was improved. Community members participated in the FGD reported they learned very important information about disease prevention. A female community discussant explained, “We learned from Urban Health Extension Professionals how much we are affecting our health and our children by simply affecting our environment”.

Regarding the lessons in family health, in all of the kebeles, Urban Health Extension Professionals reported they are providing family planning services (provision of oral contraceptives orinjectables) regularly. Regarding the other services on family health sessions, Urban Health Extension Professionals reported teaching the promotion and the utilization of maternal and child health services. Moreover, teachings about healthy behaviours like proper feeding habits (such as breast feeding, and supplements for babies), nutrition for pregnant women and adolescent reproductive health counselling were also reported.

From 308 households who had contacted Urban Health Extension Professionals, 252 (81.81%) reported they received health education on at least one of the packages included in family health. One hundred twenty six (40.90%) interviewees reported they received at least one service found in the family health package. The sessionsteaching about personal hygiene and environmental sanitation were planned to provide adequate information in seven areas. These include proper and safe excreta disposal, proper and safe solid and liquid waste management, water supply safety measures, food hygiene and safety measure, healthy home environment and personal hygiene.

From 308 households, 293 (95.12%) reported they received health education on at least one of the packages included in personal hygiene and environmental sanitation. Two hundred eighty eight (93.50%) received support in construction of sanitation facilities. A total of 275 households reported using different kinds of liquid waste disposal mechanism. From this group, 177 (64.4%) reported receiving advise and/or support from Urban Health Extension Professionals. A total of 103 households reported availability of hand washing facility near to their latrine. From this group, 52 (50.5%) reported they received advice and/or support from Urban Health Extension Professionals.

Qualitative data also supported this finding. Participants across the group felt the program helped households use hand washing facility near to their latrines; separate liquid waste disposal pits; use clean cooking practices, keep drinking water free from contamination and mange clean environment.

The other key service areas were first aid, emergency and referral. According to the implementation guideline, first aid and emergency services include attending precipitatous deliveries, fever management in under 5 year old children, managing minor wounds, bleeding and allergy management. None of Urban Health Extension Professionals in the study area started providing first aid and emergency services due to lack of supplies. The only activity in this package the Urban Health Extension Professionals reported was referral.

From 308 households, 47 (15.25%) reported they received help from Urban Health Extension Professionals to care for a sick person at home. Two hundred ninety one households visited the health facility for a different reason. From this group, none of them mentioned a prior Urban Health Extension Professionals contact for referral. Based on these group discussions and interviews, the major factor affecting the ability for these households to adopt and utilize healthy practices is the acceptance of the Urban Health Extension Professional. Community acceptance was also reported to be the most difficult to achieve. There was also resistance of some community members to accept home visits from Urban Health Extension Professionals, and to train as a model family. These were important factors in the ability of these households to adopt healthy practices. Urban Health Extension Professionals discussants mentioned that in beginning there were many people who were hesitant to accept their services. The following quotes are cited as examples: A participant said, “When we go to houses of rich people, they tell us that they have personal doctors and they don’t need us. When we go to the poor, they will tell us they are busy with their livelihood earnings”. Another participant said, “When I go to some of the houses, I have to growth kebele security officers otherwise no one is willing to talk to me”.

Urban health extension professionals said that community resistance sometimes rose from lack of awareness about the service. They explained that the usual community perception of health extension services was derived from the practice of giving services to rural community. Urban Health Extension Professionals explained, “Most of urban people live in unsanitary conditions that are worse than rural communities, but they still tell us that they are not rural people, therefore, don’t need health extension service”.

Similarly, community members participated in FGD said that, “If the community basically understand the Urban Health Extension Professionals purpose, I don’t think there is any reason to resist their service”.

Most of the Urban Health Extension Professionals mentioned supply problems creating resistance for some community members. The Urban Health Extension Professionals reported that lack of some supplies found within the guidelines (first aid and emergency supplies) created a problem in the delivery services. For example, Urban Health Extension Professionals indicated that some households especially, the poor, ask Insecticide-Treated Net (ITN), treatment for their children and anti-pain for minor illness. However, these activities and requests are clearly outside the objectives and purpose of the educators.. Urban Health Extension Professionals also indicated that the inability to provide a wider range of services adversely affected their credibility and community interest.

Both the community discussants and Urban Health Extension Professionals believe that the Urban Health Extension Professionals service would be more acceptable if Urban Health Extension Professionals could treat some illnesses. However, the supervisors and health centre managers did not agree with this perspective. They believe Urban Health Extension Professionals should work more on raising community awareness on the importance of preventive and promotive services rather than play a curative role in the health care delivery.

The other factors mentioned were economic and educational status of the household members. Urban Health Extension Professionals participants claimed that the degree of behavioural change and adoption of healthy practices in the community were often dependent on other societal factors. Urban Health Extension Professionals explained that economic status of households, such as lack of materials to construct sanitation facilities, provided a significant barrier to adoption of healthy household practices.

From the in-depth interviews with health center managers and supervisors, an additional factor in the acceptance of the Urban Health Extension Professionals was identified. emerged The subjective attributes of the particular Urban Health Extension Professionals, such as interest in their work and ability to communicate well, were identified as factors affected the acceptability of Urban Health Extension Professionals and furthermore, theutilization of their services. Urban Health Extension Professionals with good communication and interaction skills were reported to have built stronger ties with their community members.

Similarly, kebele administrative heads and health committee discussants identified if the Urban Health Extension Professionals had good communication skills, there was a higher demand for their services.

During their interviews, Urban Health Extension Professionals reported that institutional support from kebele officials could serve as the bridge for enhancing relationships between them and their community members. This was especially important for community members who are refusing their service. An FGD discussant from Urban Health Extension Professionals reported, “Kebele council support is very important and without it we may not have been able to enter some houses”. She added “Council members influence reluctant families to apply for some packages”.

The important role of kebele support in mobilizing the community and managing reluctant households was acknowledged by the program supervisors and health centre managers.

All the participants across the groups reported that they are witnessing progress acceptance by the people. The increasing community members’ participation in meetings called by Urban Health Extension Professionals, and the decrease in number of resistant households were mentioned as a positive indicator of progress. Urban Health Extension Professionals discussant said, “At first, most people saw the government cadres and thought we were working for political ends, but now they have at least realized that we are working for the sake of the people’s health”.

## Discussion

This study found out that 72.8% households had some service related contact with Urban Health Extension Professionals during the previous 6 months. The data also showed that none of the households’ received twice monthly home visit as per the guideline. This frequency seemed to be inadequate to bring about the expected behavioural change. On the other hand, the reported level of contact between Urban Health Extension Professionals and the population was a success of the program. Each Urban Extension Professional was given a higher number of households in their kebele to visit than was described in the initial guidelines. This made it difficult for the Urban Health Extension Professionals to visit the household the prescribed number of times per month.

Most of the educational topics discussed were family health and environmental sanitation. Other topics, such as prevention of non-communicable diseases and mental health, were discussed relatively less often. These results are similar with those found in Welkait, Ethiopia [23]. However, the guidelines allowUrban Health Extension Professionals to select appropriate educational topics for each household according to their needs. The implementation guidelines request all households in the area to graduate as a model family within the first one and half years of program implementation. According to this goal, this study found that the model family training rate was not happening at as expected. This indicates the need for a huge push to upgrade the interest of the community and the need to adjustment to local community needs.

Qualitative study indicates that acceptance of the UHES was associated with service use and adoption of the promoted practices. Similarly, experience elsewhere on community health workers found community acceptance as an important factor for greater use of services [24–26]. Primary Health Care Operations Research (PRICOR) funded studies done on more than 30 different countries to identify approaches for increasing the community’s utilization of community health works services. Their research suggests that improving acceptability of community health workers is the biggest factor affecting utilization of their services [27]. They found that when community members appreciate and accept the community-based services, they are more likely to use the services given at their local health post.

This study revealed that Urban Health Extension Professionals individual characteristics, like communication skills, affected levels of activity and the use of healthy interventions (both receiving the intervention and adhering to its implementation at home). This result is consistent with several other studies done on community health workers [24, 25, 28]. One possible explanation is that community health workers should understand the different behaviours in their communities and design appropriate responses to improve the acceptance of their services. This study also found that Urban Health Extension Professionals were unable to carry out all of their given tasks. For example, the guidelines suggest that they should be able to give focused antenatal care; attend precipitated deliveries, fever management in under five and screening of malnutrition via mid-upper arm circumference strips independently. However, due to the unavailability of the supplies, a delivery of such services by Urban Health Extension Professionals was not started. In addition, this study found that people expect a reasonable package of curative services from the Urban Health Extension Professionals. Therefore lack of some medicines and supplies according to the guidelines was perceived to affect credibility of Urban Health Extension Professionals.

This is consistent with studies done in other community health workers, Parlato and Favin [29]. In their review of 52 community health worker projects, the results implicated that if community health workers do not have a necessary supply and cannot perform their duties, they lose support and credibility from the communities they serve. The results also suggested that lack of credibility from the community could diminish acceptance and utilization of the community health workers’ service. Several other studies found that when curative care is offered by community based health services; it is generally more welcomed and appreciated by the residents [30–32].

This study also compared the Urban Health Extension Professionals functionality and the support they get to implement the guidelines [1]. One of the main finding of this study was the lack of quality workplace infrastructure (e.g. buildings and equipment). While there does not seem to be any guiding principles on the specification of health posts, the quality seemed to negative affect the Urban Health Extension Professionals ability to support their community. These results were consistent with other studies done on the rural health extension program [33, 34]. Issues, such as equipment and supplies, have also been identified as a limiting factor in community health worker programs in different international studies [23, 35–37].

With regard to staffing, the implementation guideline states that one UHEP gives service for 500 households. However, within eight out of the nine kebeles in the study area, the Urban Health Extension Professionals served a higher number of households. This was found to influence the ability of the UHEP to provide services like conducting regular and frequent home visits. This higher ration of households to UHEPs created a barrier to service use.

The implementation guideline also requests for service delivery at community, school and youth centre level. Literature on community health workers suggest they need to extend their services to workplaces, schools, youth organizations and among women’s associations. The extended services help to develop the capacity of these various groups by taking responsibility for their own health and the health of their communities [35, 37–39]. However, the finding of this study shows that with respect to the site of service delivery, Urban Health Extension Professionals started delivery of service only at the household and community level. One other potential flaw in this study is recall bias, as the 6 month recall history may be too difficult for participants to remember.

## Conclusions

The introduction of Urban Health Extension Professionals contributed to the household level extension of health care system and the growing primary health service coverage to the urban population. There was relatively higher rate of contact by Urban Health Extension Professionals than previously existing health service. The rate of contact and the type of service provided by these Urban Health Extension Professionals was acceptable according to the Ethiopian implementation guideline.

Though utilization of services is high, contact was initiated by HEWs, not households. Therefore, in the future the government should work in increasing house hold initiated service utilization than HEWs initiated service utilization. This study also showed that the communication skills of Urban Health Extension Professionals, kebele (neighbourhood) council support and logistics were found to be essential factors affecting the acceptance and utilization of these services. The implementing partners should provide appropriate logistic supplies and training to Urban Health Extension Professionals to increase utilization and acceptability by the community.

## Additional file

Additional file 1: English version Questionnaire. A. Household Survey questionnaire. Part I: Socio-demographic and socioeconomic data. Part II: General health care utilization/and preference. PART IV: Coverage and utilization of UHEPs. PART V: General satisfaction. B. Topic Guide Focus group discussion English version. For Health Extension Professionals. For health committee members. For community members. C Topic Guide for key informants. For Urban health extension professionals. For Health Extension Supervisors. For health centre managers. For town health department. For kebele administrative head. (DOCX 60 kb)

## Abbreviations

CHWs: Community Health Workers
FGD: Focus Group Discussion
FMOH: Federal Ministry of Health
HEP: Health Service Extension Programme
HEWs: Health Extension Workers
ITN: Insecticide-Treated Net
PHC: Primary Health Care
PRICOR: Primary Health Care Operations Research
UHEP: Urban Health Extension Program
UHES: Urban Health Extension Services
