# Supplementary material for: Urban Health Extension Services Utilization in Bishoftu Town, Oromia Regional State, Central Ethiopia
Source: BMC Health Serv Res. 2017 Mar 14;17:195. doi: 10.1186/s12913-017-2129-z (PMC5348781; doi:10.1186/s12913-017-2129-z)
Supplement: Additional file 1: — English version Questionnaire. A. Household Survey questionnaire. Part I: Socio-demographic and socioeconomic data. Part II: General health care utilization/and preference. PART IV: Coverage and utilization of UHEPs. PART V: General satisfaction. B. Topic Guide Focus group discussion English version. For Health Extension Professionals. For health committee members. For community members. C Topic Guide for key informants. For Urban health extension professionals. For Health Extension Supervisors. For health centre managers. For town health department. For kebele administrative head. (DOCX 60 kb) [file 12913_2017_2129_MOESM1_ESM.docx]

# English version Questionnaire

## Household Survey questionnaire

| Part I:Socio-demographic and socioeconomic data | | | |
| --- | --- | --- | --- |
| NO. | QUESTIONS | CODING CLASSIFICATION | Go to |
| 101 | Status of respondent in the family? (read out the answers) | 1=Household head 2= Spouse |  |
| 102 | Respondent age in years (write the age in the corresponding box) | 1 =______________ |  |
| 103 | What is your marital status? | 1=Single 2=Married  3=Separated 4=Divorced 5=Widowed |  |
| 104 | Religion | 1. Orthodox Christian 2. Muslim 3. Catholic 4. Protestant 5. Other (specify) |  |
| 105 | Ethnicity | 1. Amhara 2. Oromo 3. Tigray 4. Gurage 5. Others (specify) |  |
| 106 | What is your level of education? | 1=Illiterate 2= Able to read and write without formal education  3=mention the highest grade completed  _________________ |  |
| 107 | Number of household members? read out the answers) (write the total number of persons in that household for each age group) | 1. Below 5 years:_________ 2. 6-15 years :___________ 3. 16-49 years : ___________ 4. 50-60 years : ___________ 5. above 60 years:_________ |  |
| 108 | Highest educational level in the household? | 1=Illiterate 2= Able to read and write without formal education  3=mention the highest grade completed  _________________ |  |
| 109 | Occupational status of household members  Select from the following list   1. Farmer 2. Housewife 3. Merchant 4. Government employee 5. Self employ (vendor) 6. private organization /NGO employee 7. Daily labourer 8. Student 9. Other (Specify | House hold member  1=household head  ---------------------  2=spouse (if the household is male headed)  --------------------- |  |
| 110 | How much total income did you and your family receive monthly, not just from wages or salaries but also from all sources? (Estimated in Birr) | 1 =______________ |  |
| 111 | How much money do you spend monthly (for food, clothes, schooling, health and all others) (Estimated in Birr) | 1 =______________ |  |
| 112 | Status of the house? | 1. Owned 2. Rented 3. Other |  |
| 113 | What kind of toilet facility does most members of your household use? | 1=Private latrine  2=Communal latrine  3= Public latrine  4= No facility/bush/field  5=Other(specify) ________________ | 119 |
| 114 | Have you received any advice and/or support from other bodies outside the family to construct the latrine? | 1=Yes  2= No | 116 |
| 115 | If the answer for Q114 is yes from whom you received the advice/support from? | 1= UHEPS  2= VCHWs  3= Model families  4= Others |  |
| 116 | Do your household have any hand washing facility near to the latrine? | 1=Yes  2=No | 119 |
| 117 | Have you received any advice and/or support from other bodies outside the family to construct the hand washing facility? | 1=Yes  2=No | 119 |
| 118 | If the answer for Q117 is yes, from whom you received the advice/support from? | 1=UHEPS  2=VCHWs  3=Model families  4=Others |  |
| 119 | What kind of facility /method the household use of to dispose liquid waste? | 1. Collection tank 2. Sewer line 3. Liquid waste disposal pit 4. Latrine 5. None /open field |  |
| 120 | If the answer for Q 119 is 3 Have you received any advise and/or support from other bodies outside the family to construct liquid waste disposal pit | 1=Yes  2=No | 123 |
| 121 | If the answer for Q119 is yes from whom you received the advice/support from | 1=UHEPS  2=VCHWs  3=Model families  4=Others |  |
| 122 | What kind of facility /method the household use of to dispose Trash (garbage) ? | 1. Garbage collection system 2. Dumping 3. Burn 4. Road side 5. Other (specify) |  |

| Part II :General health care utilization /and preference | | | |
| --- | --- | --- | --- |
| NO. | QUESTIONS | CODING CLASSIFICATION | GO TO |
| 201 | Has any member of your household received any  Services at a health facility at any time in the past 6 months? | 1=YES  2 =NO | 204 |
| 202 | For what kind of service do you use the health facility?  (circle the service type used)  ( Write the number of the type of health facility the service used from in front of the service)   1. Went government health centre directly 2. Went government hospital directly 3. from private clinics /hospital 4. from drugs vender / pharmacy 5. from traditional healer 6. other specify | 1. Treatment for a sick child? 2. Treatment for illness/emergency for older member of the family? 3. For medical consultation about general health? 4. Immunization/growth monitoring? 5. Family planning services? 6. Prenatal/postnatal/delivery care? 7. Others (specify) |  |
| 203 | For any of the services you used have you been referred by health extension professionals? | 1=YES  2 =NO |  |
| 204 | Generally how do you see yours and your families general health status | 1. Very good 2. Good 3. Normal 4. Bad 5. Very bad |  |

| PART III : Awareness/attitude questions | | | |
| --- | --- | --- | --- |
| NO. | QUESTIONS | CODING CLASSIFICATION | GOTO |
| 301 | Have you heard about health extension professional? | 1 = yes  2 = No |  |
| 302 | If yes, from where did you hear about health extension professional? | 1 = From urban health extension professionals  2 = from volunteer community health worker  3 = from radio or television  4 = from friend or relative  5 = from other members of the community |  |
| 303 | Do you know the health extension professional assigned in this kebele? | 1=yes 2= No | 305 |
| 304 | How do you / your family see the start of UHES in your kebele? | 1=Strongly agree 2=Agree 3=Neutral 4=Disagree 5=Strongly disagree |  |
| 305 | Which type of service could you receive from the urban health extension professionals? | 1. Advice on health and health related issue 2. Information/counselling during illness 3. First aid /Emergency service 4. Growth monitoring 5. Family planning service 6. FANC 7. HIV counsling and testing 8. Measure vital signs like blood pressure   10=Other (specify)  11= I don’t know |  |
| How much do you agree or disagree with the statement below? | | | |
| 306 | UHEPs services are practical options to improve the health of you and your families. | 1=Strongly agree 2=Agree 3= Uncertain 4=Disagree 5=Strongly disagree |  |
| 307 | UHEPs services are practical options to improve the health of your community in general. | 1=Strongly agree 2=Agree 3=Uncertain 4=Disagree 5=Strongly disagree |  |
| 308 | your household should have contact the UHEPs On regular basis | 1=Strongly agree 2=Agree 3= Uncertain 4=Disagree 5=Strongly disagree |  |
| 309 | You recommend using this service to a friend or relative household. | 1=Strongly agree 2=Agree 3= Uncertain 4=Disagree 5=Strongly disagree |  |
| 310 | Do you know mode of service delivery that you or other members of the household will be able to get service from UHEPs? | 1=Yes 2= No | 400 |
| 311 | If yes, can you mention some? | 1. house visit by UHEPs 2. in health post/kebele 3. Other (specify) | 312  313 |
| 312 | How much do you agree or disagree with the statement below?  The service being delivered through house-to-house visit by UHEPs. | 1=Strongly agree 2=Agree 3= Uncertain 4=Disagree 5=Strongly disagree |  |
| 313 | How much do you agree or disagree with the statement below?  The UHES being delivered in your kebele health  Post/ kebele compound. | 1=Strongly agree 2=Agree 3= Uncertain 4=Disagree 5=Strongly disagree |  |

| PART IV : Coverage and utilization of UHEPs | | | | |
| --- | --- | --- | --- | --- |
| NO. | QUESTIONS | | CODING CLASSIFICATION | GO TO |
| 400 | Have you or any member of your household any service-related contact with UHEPs in the previous 6 months? | | 1=Yes 2= No |  |
| 401 | If the answer for Q 400 is, yes, how many times you did or any member of your household had any service-related contact with UHEPs in the previous 6 months? | | 1= |  |
| 402 | If the answer for Q 400 is yes when was the last time that the contact has been done? | | 1 = Less than one month ago 2 = Two months ago 3 = Three months ago 4= More than three months ago  5= More than four months ago  6= More than five months ago |  |
| 403 | How much satisfied are you with your frequency of contact with UHEPs | | 1. Very satisfied 2. Satisfied 3. Neither satisfied nor dissatisfied 4. Not very satisfied 5. Not satisfied at all |  |
| 404 | If the answer for Q 400 is, yes, where was the contact been done? | | 1. at home 2. At health post/kebele 3. at community meetings 4. At religious institutions 5. At cultural institutions(edir,ikub) 6. at work place 7. at school 8. other places (specify) | 405  417- 419  420-426  428-433  428-433  428-433  428-433 |
| 405 | The answer for Q 404 is one how was the home contact made? | | 1= House visit by the UHEPS  2= Had UHEPS come to home  3= others( specify ) | 406-413  414-416 |
| 406 | If the answer for Q 405 is one, how many times did the UHEPs visited your house in the last six months? | | 1= |  |
| 407 | If the answer for Q 405 is one When was the last time that a health extension professional visited your home? | | 1 = Less than one month ago 2 = Two months ago 3 = Three months ago 4= More than three months ago  5= More than four months ago  6= More than five months ago |  |
| 408 | When the health extension professional visited your home, what did she do? ( read out the answers)  (more than one answers accepted) | | 1, Yes 2= No  1 = Give health education 2 = helped care for sick person  3 = Administered first aid emergency  4= Give family planning service  5 = Looked after sick baby and baby mother  6 = Give HIV/AIDS counselling and testing service  7= Give growth monitoring service.  8= Attend precipitated labour  9= Others (specify) | 409 |
| 409 | What specific health education information did you received from health extension worker?( read out the answers)  (more than one answers accepted) | | 1, Yes 2= No  1 = Immunization  2 = nutrition  3 = food safety management  4 = Pregnancy care/delivery plan  5 = HIV/AIDS  6= TB/ leprosy  7= Malaria prevention  8 = Waste management  9 = Latrine construction/use  10 = Safe / clean water  11= Family planning  12= Adolescent reproductive health  13= non communicable disease ( cancer, diabetes ,hypertension )  14= Mental health  15= Housing and environmental sanitation  16=others (specify ) |  |
| 410 | Do you ask education or any assistant related to health you need during her visits? | | 1 = Yes  2 = No |  |
| 411 | During her visits was the HEP easy to understand? | | 1 = Yes  2 = No |  |
| 412 | How satisfied are you with your household interaction with UHEPs? | | 1. Very satisfied 2. Satisfied 3. Neither satisfied nor dissatisfied 4. Not very satisfied 5. Not satisfied at all |  |
| 413 | How help full was the service to this service to you and your family? | | 1. Very useful 2. Useful 3. Uncertain 4. Not very useful 5. Not useful at all |  |
| 414 | If the answer for Q 405 is two what is the most important reason for calling a visit from the UHEPs ? | | 1. for consultation on general health 2. a member of the family was sick 3. for growth monitoring 4. for family planning 5. for precipitated deliveries 6. For HIV counselling and testing 7. to get vital sign checked 8. for first aid/emergency service 9. other reason(specify):__________ |  |
| 415 | How satisfied are you with your household interaction with UHEPs? | | 1. Very satisfied 2. Satisfied 3. Neither satisfied nor dissatisfied 4. Not very satisfied 5. Not satisfied at all |  |
| 416 | How help full was the service to this service to you and your family? | | 1. Very useful 2. Useful 3. Uncertain 4. Not very useful 5. Not useful at all |  |
| 417 | If the answer for Q 404 is two What is the most important reason for visiting HEPs?  ( more than one answer is accepted ) | | 1. member of the family was sick 2. for consultation on general   health   1. growth monitoring 2. adolescent reproductive Health services 3. Went for family planning 4. Went for ANC 5. HIV counselling and testing 6. to get vital sign checked 7. for firs aid/ emergency service 8. other reason(specify):__________ |  |
| 418 | Have you get the service you need during the visit ? | | 1 = Yes  2 = No |  |
| 419 | If the answer for Q 417 is, yes how much are you satisfied with the service you received? | | 1. Very satisfied 2. Satisfied 3. Neither satisfied nor dissatisfied 4. Not very satisfied 5. Not satisfied at all |  |
| 420 | During the visits to the health post, were the UHEPs easy to understand? | | 1 = Yes  2 = No |  |
| 421 | If the answer for Q 403 is three, Did the community meeting have been organized by the UHEPs? | | 1 = Yes  2 = No  3= don’t know |  |
| 422 | Did the health extension professionals give health education on the community meeting? | | 1 = Yes  2 = No | 427 |
| 423 | If the answer for Q 421 is yes, What specific health education information did you received from health extension professionals? ( read out the answers)  (more than one answers accepted) | | 1, yes 2=no  1 = Immunization  2 = nutrition  3 = food safety management  4 = Pregnancy care/delivery plan  5 = HIV/AIDS  6= TB/ leprosy  7= Malaria prevention  8 = Waste management  9 = Latrine construction/use  10 = Safe / clean water  11= Family planning  12= Adolescent reproductive health  13= non communicable disease ( cancer, diabetes ,hypertension )  14= Mental health  15= Housing and environmental sanitation  16=others (specify ) |  |
| 424 | How satisfied are you with your house hold interaction with UHEPs | | 1. Very satisfied 2. Satisfied 3. Neither satisfied nor dissatisfied 4. Not very satisfied 5. Not satisfied at all |  |
| 425 | How help full was the service to this service to you and your family? | | 1. Very useful 2. Somewhat Useful 3. Uncertain 4. Not very useful 5. Not useful at all |  |
| 426 | During the meeting was the HEP easy to understand? | | 1 = Yes  2 = No |  |
| 427 | Do you ask education or any assistant related to health you need during the community meeting? | | 1 = 1 = Yes ,we got what I asked for  2=yes ,but we didn’t got what we asked for  3 = No |  |
| 428 | If the answer for Q 403 is 4/5/6/7, Did the health extension professionals give health education? | | 1 = yes  2 = No | 433 |
| 429 | What specific health education information did you received from health extension professionals? (read out the answers)  (more than one answers accepted) | | 1, yes 2=no  1 = Immunization  2 = nutrition  3 = food safety management  4 = Pregnancy care/delivery plan  5 = HIV/AIDS  6= TB/ leprosy  7= Malaria prevention  8 = Waste management  9 = Latrine construction/use  10 = Safe / clean water  11= Family planning  12= Adolescent reproductive health  13= non communicable disease ( cancer, diabetes ,hypertension )  14= Mental health  15= Housing and environmental sanitation  16=others (specify ) |  |
| 430 | How satisfied are you with your house hold interaction with UHEPs | | 1. Very satisfied 2. Satisfied 3. Neither satisfied nor dissatisfied 4. Not very satisfied 5. Not satisfied at all |  |
| 432 | How help full was the service to this service to you and your family? | | 1. Very useful 2. Somewhat Useful 3. Uncertain 4. Not very useful 5. Not useful at all |  |
| 433 | During the contact was the HEP easy to understand? | | 1 = Yes  2 = No |  |
| 433 | Do you ask education or any assistant related to health you need during the contact? | | 1 = Yes ,we got what I asked for  2=yes ,but we didn’t got what we asked for  3 = No |  |
| 434 | Have you heard about a model family? | | 1 = yes  2 = No | 439 |
| 435 | If yes, from where did you hear about model family? | | 1 = from urban health extension professionals  2 = from community health workers  3 = from member of my community  4 = from friend or relative  5 = from radio or television  6 = from other source (specify) |  |
| 436 | Has your family participated in a model family training? | | 1 = yes  2 = No | 438 |
| 437 | If yes, did you family finish the model family training & got certificate? | | 1 = yes, and showed certificate  2 = yes, did not show certificate  3 = Presently in training  4 = Did not finish the training, drop out |  |
| 438 | In what year was the certificate issue? Write the month and year? | | 1=Month.____Year._2 / 0 /__/___/ |  |
| 439 | Have your family been asked to participate in model family training? | | 1=Yes but never interested to participate  2=Yes but we have no time for the training  2 = No |  |
| 440 | Are there services that you have needed but have been unable to find from UHES? | | 1. Yes  2. No | 441 |
| 441 | Can you mention what services those were? | | 1. ________________________  2. ________________________  3. ________________________ |  |
| VHWs | | | | |
| 442 | Have you heard about volunteer community health workers? | 1 = yes  2 = No | | 501 |
| 443 | From where did you hear about volunteer community health workers? | 1 = from health extension worker  2 = from volunteer community health worker  3 = from community members  4= from relative or friend  5= from radio or television  6=others (specify ) | |  |
| 444 | Have any community health workers visited you home in the last three months? | 1 = yes  2 = No | | 501 |
|  | What kind of information did the community health worker provide when she/he visited your home?(read out the answers)  (More than one answer is acceptable ) | 1, yes 2=no  1 = Immunization  2 = nutrition  3 = food safety management  4 = Pregnancy care/delivery plan  5 = HIV/AIDS  6= TB/ leprosy  7= Malaria prevention  8 = Waste management  9 = Latrine construction/use  10 = Safe / clean water  11= Family planning  12= Adolescent reproductive health  13= non communicable disease ( cancer, diabetes ,hypertension )  14= Mental health  15= Housing and environmental sanitation  16=others (specify ) | |  |

| PART V : General satisfaction | | | |
| --- | --- | --- | --- |
| NO. | QUESTIONS | CODING CLASSIFICATION | GO TO |
| 501 | How satisfied are you with communication ability of UHEPs? | 1. Very satisfied 2. Satisfied 3. Neither satisfied nor dissatisfied 4. Not very satisfied 5. Not satisfied at all |  |
| 502 | How satisfied are you in the usefulness of the service you received from UHEPs? | 1. Very satisfied 2. Satisfied 3. Neither satisfied nor dissatisfied 4. Not very satisfied 5. Not satisfied at all |  |
| How much do you agree or disagree with the statement below ? | | | |
| 503 | During your contacts the UHEPs, has good professional competence as a health professional? | 1=Strongly agree 2=Agree 3=Uncertain 4=Disagree 5=Strongly disagree |  |
| 504 | During the contacts it was easy to understand what the UHEP teach or advice? | 1=Strongly agree 2=Agree 3=Uncertain 4=Disagree 5=Strongly disagree |  |
| 505 | The UHEPs respect my family and me during the contact. | 1=Strongly agree 2=Agree 3= Uncertain 4=Disagree 5=Strongly disagree |  |
| 506 | The UHEPs are with no discrimination.  . | 1=Strongly agree 2=Agree 3= Uncertain 4=Disagree 5=Strongly disagree |  |
| 507 | **U**HEPs were always available when needed | 1=Strongly agree 2=Agree 3= Uncertain 4=Disagree 5=Strongly disagree |  |

I have finished the interview Thank you very much.

## Topic Guide Focus group discussion English version.

- - - 1. **for Health Extension Professionals**
- Can you tell what it is like to be urban health extension professional? What is your work like?
- How do you select homes to visit? Is there disadvantaged groups specifically targeted by your program?
- What determines how many households you will see?
- What do you think the community thinks of your work? Are there any people who refuse to use your service?
- What does the community expect/demand from you?
- Do you feel that you can satisfy their expectations/demands? How? Why?
- Can anyone tell me the thing you like the most about your work as a UHEP?
- What kinds of things don't you like about your work?
- What are some of the main problems that you face in your work?
- Can you think of any solutions for these problems?"
- Do you have anything else to suggest or comment on?
  - - 1. **for health committee members**
- How do you see the start of implementation of the program in the kebele?
- What are the potential use and challenge of the program?
- How do you see the current acceptability and service uptake by the members of the community?
- What determines the current service delivery and use? House to house visit, model family training?
- How do you see the program impact on your community? What kind? How do you know?
- What suggestions do you have for the further implementation of the program?
- Do you have anything else to suggest or comment on?
  - - 1. **for community members**
- How do you see the start of implementation of the program in the kebele?
- What are the potential use and challenge of the program?
- Can anyone tell me the thing you like best about the work of UHEPs?
- How do you see the current acceptability and service uptake by the members of the community?
- How do you see Community acceptability about the service and the UHEPs?
- What determines the community acceptability and uptake of the service availed through UHEPS?
- How do you see the program impact on your community? What kind? How do you know?
- What suggestions do you have for the further implementation of the program?
- Do you have anything else to suggest or comment on?

## Topic Guide for key informants

**1. For Urban health extension professionals**

| 001. Name of kebele: _________________________________________  002 .Name of data collector: _____________________________________________  003. Date of interview: _____ /Oct. / 2012  004. Time start:__/__:__/___ time end:__/__:_ | |
| --- | --- |
| Teams | Questions |
| Service delivery | Are you providing the full (16) package of the health extension program in this kebele?  What components are not being provided? Why not?  What component of the package is performing better and which is performing less?  What are the enablers for better performance and disablers for less performance? |
| Model family selection and training | How are families selected to be trained at a time as model families?  How is the model families training done? Number of training session held per week, days of the week session are held and time spent in hour & minutes  What are the enablers for better performance in model family training and disablers for less performance? |
| Home visitation | Do you conduct regular home visit as a part of your job in your kebele?  How many homes do you visit per week?  What determines the number of households to visit?  What activities do you perform during your home visit?  What determines the type of activity you perform?  How do you generally evaluate the house visitation? What are the enablers for better performance in house visitation and disablers for less performance? |
| Health post services | How many days in the week is the health post open?  Are the community members in this kebele aware of your working schedule at this health post? How is the community informed?  Do individuals come to the health post to receive service? |
| Community participation and acceptance | How do you involve the community members into you activities? Do people ever come to you for advice, or just to talk out their problems? If Yes, about how many different people per week? What determines the community acceptance of your service? |
| Reporting and information system | To whom do you submit your reports?  How often do you submit reports?  What kind of feedbacks do you received from those to whom you submit your reports?  Do you have standardized reporting forms? |
| Supply chain | Do you have the supplies and equipment you need to provide the Services you are expected to deliver?  How do you get your medicines and supplies?  Is there a standardized requisition form for use by you to request supplies?  What are the average time laps between requisitions for supplies and the delivery of supplies?  If you have experienced shortages or stock outs of supplies, please provide the information about the commodity or commodities? |
| Collaboration across different sectors | Is there a functional kebele health council?  How is the kebele health council involved in your activities?  How is the kebele council involved in your activities?  What are the other sectors that are collaborating with you in the health extension program? What are their levels of involvement and what role do they play? |
| Referral | Do you refer clients for health services you do not or cannot provide?  Do you complete a referral form for the client to take to the facility?  Please describe any feedback or counter referral you receive from the facility for clients you have referred? |
| Service overall assessment | What are your biggest challenges as UHEP?  What changes are needed to help you do your job better? |

**2. for Health Extension Supervisors**

| 001. Health Centre Code: ___ /___  002.Data collector’ name _____________________________________  003. Date of interview: _____ / Oct./2012  004. Time start: __/__:__/___: Time end __/___:__/_ | |
| --- | --- |
| Service delivery | Is the full (16) package of the health extension program being delivered in your supervision areas?  What do you think are the enablers for better performance?  What are disabling factors for less performance?  How can these disablers be removed to improve performance of all components of the program?  Are there any obstacles that have prevented UHP’s from serving clients and implementing the program according to the national guidelines |
| Community participation and acceptance | What provider issues have been obstacles in meeting community need?  What user issues have been obstacles in meeting the program objective?  How do you evaluate the uptake of the service by the community? How are people responding? |
| Monitoring | How is monitoring done?  What are monitored?  Have a standardized monitoring tool?  How are Data/ reports received from health post managed, processed and used? |
| Supply chain | Do the health extension professionals have the supplies and equipment they need to provide the Services they are expected to deliver?  How does the supply chain work? How do you get more supplies? How often do you get them? What form(s) do you use?  What roles do the woreda, health post and kebele health council play in the supply chain system? |
| Collaboration across different sectors | What are the other sectors that are collaborating with you in the health extension program? What are their levels of involvement and what role do they play? |
| UHES over all assessment | The Success  What determines the success  The Challenges  What is the solution |

**3. for health centre managers**

| 001. Health Centre Code: ___ /___  002.Data collector’ name _____________________________________  003. Date of interview: _____ / Oct./2012  004. Time start: __/__:__/___: Time end __/___:__/___ | |
| --- | --- |
| General about the program implementation | How the health extension program is is being implemented in your area?  What component of the package is performing better and which is performing less?  What are the enablers for better performance and disablers for less performance?  How can these disablers be removed to improve performance of all components of the program? |
| Support to Urban health extension professionals | How does the town health center support the health extension to under take their duties?  What activities do you undertake with the health extension professionals? |
| Monitoring | Who is responsible for monitoring the health extension professionals?  How is monitoring done?  What are monitored?  What do you do with information gathered from the monitoring? |
| Supply chain | Are the needed supplies available to the health extension professionals?  How does the health post get drugs and supplies to work?  What roles does the town health center play in getting supplies for health post? |
| Collaboration across different sectors | Is there collaboration across different sectors in the program implementation?  What are the other sectors that are involved in the program?  What are their levels of involvement and what role do they play? |
| Community participation and acceptance | What provider issues have been obstacles in meeting community need?  What user issues have been obstacles in meeting the program objective?  How do you evaluate the uptake of the service by the community? How are people responding? |
| UHES over all assessment | Success  What determines the success  Challenges  What is the solution |

**4. for town health department**

| 001. Data collector’ name _____________________________________  002. Date of interview: _____ / Oct./2012  003. Time start: __/__:__/___: Time end __/___:__/___ | |  |
| --- | --- | --- |
| General about the program implementation | How the health extension program is is being implemented in your town?  What component of the package is performing better and which is performing less?  What are the enablers for better performance and disablers for less performance?  How can these disablers be removed to improve performance of all components of the program? |  |
| Support to Urban health extension professionals | How does the town health department support the health extension to under take their duties?  What activities do you undertake with the health extension professionals? |  |
| Monitoring | Who is responsible for monitoring the health extension professionals?  How is monitoring done?  What are monitored?  What do you do with information gathered from the monitoring? |  |
| Supply chain | Are the needed supplies available to the health extension professionals?  How does the health post get drugs and supplies to work?  What roles does the town health department play in getting supplies for health post? |  |
| Collaboration across different sectors | Is there collaboration across different sectors in the program implementation?  What are the other sectors that are involved in the program?  What are their levels of involvement and what role do they play? |  |
| Community participation and acceptance | What provider issues have been obstacles in meeting community need?  What user issues have been obstacles in meeting the program objective?  How do you evaluate the uptake of the service by the community? How are people responding? |  |
| UHES delivery and use overall assessment | Success  What determines the success  Challenges  What is the solution |  |

**5. for kebele administrative heads**

| 001.Name of kebele: ______________________________________  002.Data collector’ name _____________________________________  003.Date of interview: _____ / _____ / 2012  004.Time interview begins: _____ _____ : Time interview ends: ______ | | |
| --- | --- | --- |
| General about the program implementation | What do you see the start urban health extension program? How is the program being implemented in your kebele?  What difference has it made in the health of the kebele residence?  What component of the package is performing better and which is performing less?  What are the enablers for better performance and disablers for less performance?  How can these disablers be removed to improve performance of all components of the program? |  |
| Support to Urban health extension professionals | How does the kebele council support the urban health extension professionals to get community to participate in the program activities?  What activities do you undertake with the urban health extension professionals?  How do you involve the Urban health extension professionals in kebele activities?  Are you playing you role in the health extension program? What exactly are you doing? |  |
| Resource allocation | How are resources provided from the kebele council to the health extension program in you kebele? |  |
| Supervision | Is there a procedure clearly known to the urban health extension professionals how to access resources from the kebele council? |  |
| Monitoring | Who is responsible for monitoring the urban health extension professionals?  If the kebele do monitoring, how often is monitoring done?  How is monitoring done?  What are monitored?  What do you do with information gathered from the monitoring? |  |
| Supply chain | Are the needed supplies available to the urban health extension professionals?  How does the health post get drugs and supplies to work?  What roles does the kebele health council play in getting supplies for health post? |  |
| Collaboration across different sectors | Is there collaboration across different sectors in the program implementation?  What are the other sectors that are involved in the program?  What are their levels of involvement and what role do they play? |  |
| Community acceptance and use | How do you see the community acceptance from your perspective?  What problems and barriers do you see with acceptability of service by the community?  How do you evaluate the uptake of service by the community?  What problems and barriers do you see with uptake of service by the community? |  |
